# Supplementary material for: The causal relationship between the human gut microbiota and pyogenic arthritis: a Mendelian randomization study
Source: Front Cell Infect Microbiol. 2024 Nov 26;14:1452480. doi: 10.3389/fcimb.2024.1452480 (PMC11629706; doi:10.3389/fcimb.2024.1452480)
Supplement: Supplementary file 1 [file DataSheet1.zip › mendelian test/ebi-a-GCST90027510.csv_leave_one_out.pdf]

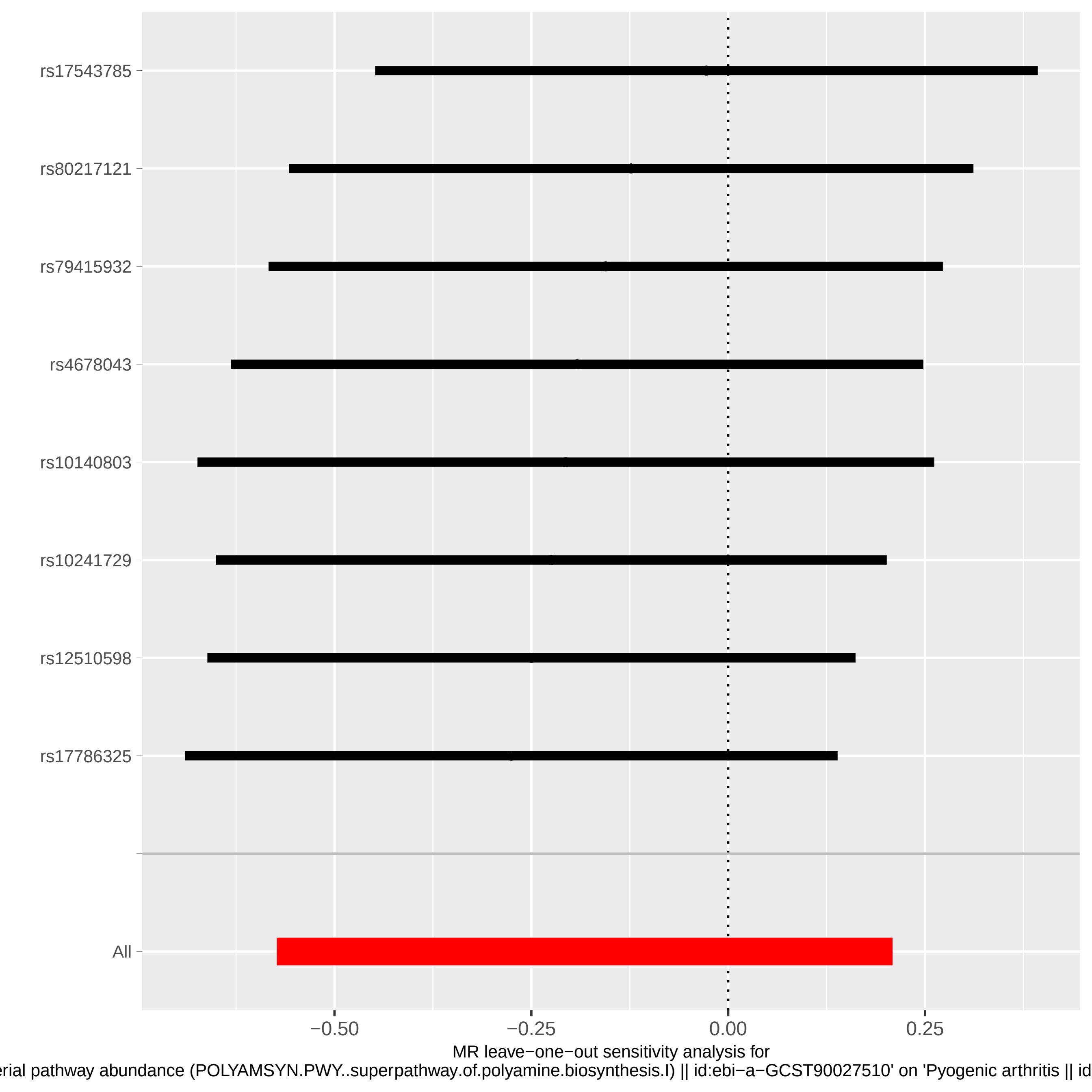

rs17543785
rs80217121
rs79415932
rs4678043
rs10140803
rs10241729
rs12510598
rs17786325
All
−0.50
−0.25
0.00
0.25
MR leave−one−out sensitivity analysis for
erial pathway abundance (POLYAMSYN.PWY..superpathway.of.polyamine.biosynthesis.I) || id:ebi−a−GCST90027510' on 'Pyogenic arthritis || id
